# Atividade Física e Redução do Comportamento Sedentário durante a Pandemia do Coronavírus

**DOI:** 10.36660/abc.20200238

**Published:** 2020-06-29

**Authors:** Francisco José Gondim Pitanga, Carmem Cristina Beck, Cristiano Penas Seara Pitanga

**Affiliations:** 1 Universidade Federal da Bahia SalvadorBA Brasil Universidade Federal da Bahia (UFBA), Salvador, BA – Brasil; 2 Instituto Federal de Santa Catarina PalhoçaSC Brasil Instituto Federal de Santa Catarina, Palhoça, SC – Brasil; 3 Universidade Católica do Salvador SalvadorBA Brasil Universidade Católica do Salvador, Salvador, BA – Brasil

**Keywords:** Pandemia, Coronavirus, Exercício, Atividade Física, Atividades de Lazer, Tempo de Tela, Dinâmica Populacional, Fenômenos Fisiológicos Cardiovasculares

## Introdução

A pandemia do novo coronavírus, recentemente declarada pela Organização Mundial da Saúde,^1^ levou diversas secretarias municipais e estaduais de saúde a publicar documentos propondo o fechamento dos diversos espaços destinados a prática da atividade física. Além disto, o Ministério da Saúde^2^ elaborou um manual com diversas ações para evitar a disseminação da doença, além de tomar decisões sugerindo o isolamento social recomendando que as pessoas permanecessem em casa. Todas essas medidas fizeram com que a população brasileira passasse a ter dificuldades para a prática de atividade física.

Por outro lado, a literatura é consistente quanto ao fornecimento de evidências sobre os diversos benefícios proporcionados pela atividade física à saúde,^3^ principalmente ao sistema cardiovascular/metabólico^4^ e imunológico.^5^ Mais recentemente, a literatura passa a apresentar evidências de que não é apenas a prática regular da atividade física que tem relação com a saúde, mas também a redução do comportamento sedentário, ou seja, o tempo que permanecemos sentados, deitados ou reclinados durante o dia, excetuando-se as horas de sono.^6^

Desta forma, parece ser muito importante a necessidade da continuidade da prática de atividade física mesmo durante a pandemia do novo coronavírus, porém medidas devem ser observadas para que essa prática possa ser considerada segura. Ressalta-se que mesmo na cidade de Wuhan, China, epicentro inicial da doença, as pessoas foram recomendadas a dar continuidade a prática de atividade física mesmo dentro de casa.^7^ Além disso, torna-se importante que a população seja esclarecida sobre a necessidade da redução do comportamento sedentário durante o período de isolamento social.

Assim, os objetivos deste ponto de vista são ressaltar a importância e propor sugestões para continuidade da prática de atividade física e redução do comportamento sedentário durante a pandemia do novo coronavírus no Brasil.

### Importância da prática de atividade física e benefícios para a saúde

#### Atividade física e saúde cardiovascular e metabólica

Os benefícios da atividade física regular sobre a saúde cardiovascular/metabólica são amplamente divulgados na literatura há bastante tempo. A atividade física apresenta associação inversa com níveis pressóricos, diabetes, alterações lipídicas e risco de doença arterial coronariana e outros eventos cardiovasculares.^4 , 8^

Com relação a duração e intensidade da atividade física necessária para que os benefícios possam ocorrer, recente publicação sugeriu que atividades na intensidade modera/vigorosa com duração de 180 a 300 minutos por semana para homens e na intensidade moderada/vigorosa com duração de 150 a 300 minutos por semana para mulheres seriam as mais adequadas para promover os benefícios para a saúde cardiovascular e metabólica.^9^ Estas recomendações estão alinhadas com os principais guias sobre atividade física, publicados por organizações internacionais.

#### Atividade física e sistema imunológico

O sistema imunológico é um importante mecanismo de defesa do nosso corpo capaz de reconhecer e eliminar uma série de micro-organismos invasores. A primeira linha de defesa é composta por leucócitos (neutrófilos, eosinófilos, basófilos, monócitos), células natural *killer* , proteínas de fase aguda e enzimas. A segunda linha de defesa é composta por linfócitos T e B e por imunoglobulinas.^5^

A prática da atividade física modula a quantidade destas substâncias no nosso organismo tanto para mais, quanto para menos e a sua magnitude depende da intensidade e duração da atividade.

Com relação aos leucócitos, por exemplo, durante a prática da atividade física existe um aumento na sua concentração, que é reduzida imediatamente após a prática dos exercícios físicos, principalmente após exercícios de longa duração e intensidade elevada que podem provocar imunossupressão em virtude da teoria da “janela aberta” quando verifica-se depressão no sistema imunológico após exercício extenuante, deixando o organismo mais suscetível a vírus e bactérias por um período de 3 a 72 horas. Ressalta-se que nas atividades de intensidade leve a moderada e com duração não prolongada o período de duração da imunossupressão é bem mais curto.^5^

#### Importância da redução do comportamento sedentário e saúde cardiometabólica

O comportamento sedentário é definido como atividades caracterizadas por baixo gasto energético, não excedendo 1,5 equivalentes metabólicos que incluem comportamentos específicos de sentar, reclinar ou deitar, para ler, estudar, assistir televisão, usar o computador, entre outras, excetuando-se as horas sono.^6^

Recente publicação demonstrou que a redução do comportamento sedentário estava associada com efeitos benéficos para diversas variáveis que representam a saúde cardiometabólica em adultos.^10^ Neste mesmo estudo os autores demonstraram, também, que quando a redução do comportamento sedentário estava associada a prática regular de atividade física os benefícios eram maximizados.

## Sugestões para continuidade da prática de atividade física durante a pandemia do novo coronavírus

### Locais para a prática da atividade física

Considerando que o Brasil é um país de dimensões continentais é importante o acompanhamento das decisões publicadas pelas secretarias estaduais/municipais de saúde e pelo Ministério da Saúde quanto as restrições e acesso às academias, clubes, clínicas e outros espaços públicos destinados a prática de atividades físicas/exercícios físicos.

No caso destes espaços estarem fechados para os usuários, a atividade física deverá ser mantida, quando possível, em ambientes abertos. Neste caso as pessoas devem priorizar as atividades feitas individualmente, sempre evitando aglomerações ou até mesmo pequenos grupos. Se todas as possibilidades citadas anteriormente estiverem com restrições a atividade física deve ter continuidade em casa, preferencialmente com auxílio de procedimentos tecnológicos, tais como vídeos com séries de exercícios, aplicativos e orientação profissional on-line.

### Tipos de atividade física/exercício físico

Quando a prática da atividade física puder ser realizada ao ar livre sugere-se atividades aeróbicas, especialmente realizadas individualmente, evitando-se aglomerações. Deve-se evitar, neste momento, a prática de esportes coletivos, mesmo que realizada em pequenos grupos.

No caso da atividade física ter que ser realizada em casa sugere-se exercícios de fortalecimento muscular (agachamentos, flexões, abdominais, entre outros), alongamentos, exercícios de equilíbrio e subida/descida de escadas, de preferência com auxílio de procedimentos tecnológicos, tais como vídeos com séries de exercícios, aplicativos e orientação profissional on-line. Ressalta-se, ainda, a importância do aumento da atividade física doméstica, ou seja, faxinas de modo geral, lavar louças, lavar e passar roupas, entre outras.

### Intensidade da atividade física

Durante a pandemia do coronavírus no Brasil recomenda-se que a intensidade dos exercícios físicos seja de leve a moderada, já que intensidades muito altas podem causar imunossupressão mais acentuada com necessidade de mais tempo para recuperação.

### Duração da atividade física

Durante a pandemia de coronavírus no Brasil recomenda-se que a duração de cada sessão de exercícios seja de aproximadamente 30 a 60 minutos por dia. Sugere-se que o tempo total não seja muito prolongado em função da depressão causada no sistema imunológico com período de recuperação mais prolongado.

### Sugestões para redução do comportamento sedentário durante a pandemia do novo coronavírus

Considerando que além da prática regular de atividade física é muito importante a redução do comportamento sedentário, sugere-se:

1. Reduzir os comportamentos sedentários para o máximo de 6 a 8 horas acumuladas durante o dia
2. Reduzir para o máximo de 2 a 4 horas sentado em frente a tela durante o dia.
3. Procurar fazer a maior quantidade de interrupções/pausas no tempo sentado, ou seja, a cada hora sentado, ficar em pé por pelo menos 5 minutos.

## Considerações finais

As evidências com base nos estudos consultados confirmam a importância da continuidade da prática de atividade física, durante a pandemia do novo coronavírus, na intensidade/duração leve a moderada, preferencialmente em ambiente abertos, ou mesmo dentro de casa. Além disto, é muito importante que se enfatize também a redução do comportamento sedentário, ou seja o tempo que ficamos sentados, deitados ou reclinados em frente a televisão, computador e semelhantes, excetuando-se as horas de sono.
