# Supplementary material for: Atividade Física e Redução do Comportamento Sedentário durante a Pandemia do Coronavírus
Source: Arq Bras Cardiol. 2020 Jun 29;114(6):1058–60. [Article in Portuguese] doi: 10.36660/abc.20200238 (PMC8416117; doi:10.36660/abc.20200238)
Supplement: Translation

# Translation: Physical Activity And Reducing Sedentary Behavior During The Coronavirus Pandemic

**Authors:** Francisco José Gondim Pitanga, Carmem Cristina Beck, Cristiano Penas Seara Pitanga

**Affiliations:** 1 Brazil Universidade Federal da Bahia (UFBA), Salvador, BA – Brazil; 2 Brazil Instituto Federal de Santa Catarina, Palhoça, SC – Brazil; 3 Brazil Universidade Católica do Salvador, Salvador, BA – Brazil

**Keywords:** Pandemics, Coronavirus, Exercise, Physical Activity, Leisure Activities, Screen Time, Population Dynamics, Cardiovascular Physiological Phenomena

## Introduction

The novel coronavirus pandemic recently declared by the World Health Organization^1^ has led several municipal and state health departments to issue decrees closing different spaces intended for practicing physical activity. Furthermore, the Ministry of Health^2^ has prepared a manual with several initiatives to prevent the spread of the disease, in addition to making decisions suggesting social isolation and recommending that people remain at home. All of these measures have made it more difficult for the Brazilian population to practice physical activity.

On the other hand, the literature has consistently provided evidence regarding the diverse health benefits that physical activity promotes,^3^ especially to the cardiovascular/metabolic^4^ and immunological system.^5^ More recently, the literature began to show evidence that health is related not only to regular practice of physical activity, but also to reduced sedentary behavior, in other words, time spent sitting, lying down, or reclining during the day, excluding sleep hours.^6^

Accordingly, there is an important need to continue practicing physical activity during the novel coronavirus pandemic; however, some measures should be observed in order to keep this practice safe. It is worth emphasizing that even in the city of Wuhan, China, the initial epicenter of the disease, it was recommended that people continue practicing physical activity inside their homes.^7^ Furthermore, it has become important that the population be informed regarding the need to reduce sedentary behavior during this period of social isolation.

Thus, the objectives of this viewpoint are to emphasize the importance of these issues and to propose suggestions for continuing practice of physical activity and reducing sedentary behavior during the novel coronavirus pandemic in Brazil.

### The important of practicing physical activity and health benefits

#### Physical activity and cardiovascular and metabolic health

The benefits of regular physical activity to cardiovascular/metabolic health have been widely published in the literature for a long time. Physical activity has been shown to be inversely associated with blood pressure levels, diabetes, lipid alterations, and other cardiovascular events.^4 , 8^

With respect to duration and intensity of physical activity required for these benefits to occur, a recent publication suggested that 180 to 300 minutes of moderate to intense activity weekly for men and 150 to 300 minutes of moderate to intense activity for women would be the most adequate measure for promoting cardiovascular and metabolic health benefits.^9^ These recommendations are in agreement with the main physical activity guidelines published by international organizations.

#### Physical activity and the immune system

The immune system is an important defense mechanism of the body. It is capable of recognizing and eliminating a series of invasive microorganisms. The first line of defense is made up of leukocytes (neutrophils, eosinophils, basophils, monocytes), which are natural killer cells, acute-phase proteins, and enzymes. The second line of defense is made up of T and B lymphocytes and imunoglobulins.^5^

Practice of physical exercise modulates the quantity of these substances in the organism, both increasing and decreasing, and the magnitude depends on the intensity and duration of physical activity.

In relation to leukocytes, for example, during practice of physical activity, their concentration increases, and it is immediately reduced following physical practice, especially after exercise with long duration and high intensity that may lead to immunosuppression, according to the theory of the “open window,” when there is a depression in the immune system after strenuous exercise, leaving the organism more susceptible to bacteria for a period of 3 to 72 hours. It is worth stressing that the period of immunosuppression is much shorter after light to moderate exercise without prolonged duration.^5^

#### The importance of reducing sedentary behavior and cardiometabolic health

Sedentary behavior is defined as activities characterized by low energy expenditure, not exceeding 1.5 metabolic equivalents, including the specific behaviors of sitting, reclining, or lying down to read, study, watch television, use the computer, etc., excluding sleep hours.^6^

A recent publication has demonstrated that reduced sedentary behavior is associated with beneficial effects on diverse variables that represent cardiometabolic health in adults.^10^ In this same study, the authors also demonstrated that, when the reduction in sedentary behavior was associated with regular practice of physical activity, the benefits were maximized.

## Suggestions for continuing practice of physical activity during the novel coronavirus pandemic

### Places for practicing physical activity

Considering that Brazil is a country of continental dimensions, it is important to accompany decisions published by state or municipal health departments and by the Ministry of Health regarding restricted access to gyms, clubs, clinics, and other spaces intended for practicing physical activity and exercise.

In the event that these spaces are closed to the public, physical activity should, whenever possible, continue in open-air environments. In this case, people should prioritize individual activities, always taking care to avoid crowds or even small groups of people. If these conditions are restricted, physical activity should be continued at home, preferably with the assistance of technology, such as exercise videos, applications, or professional guidance online.

### Types of Physical Activity/Exercise

When it is possible to practice physical activity outdoors, aerobic activities are recommended, especially individual activities; crowds should be avoided. At this moment, it is necessary to avoid practicing group sports, even in small groups.

In the event that it is necessary to exercise at home, muscle-strengthening exercises (squats, push-ups, sit-ups, and others), stretches, balancing exercises, and climbing up and down stairs are recommended, preferably with the assistance of technology, such as exercise videos, applications, and professional guidance online. It is, furthermore, worth emphasizing the importance of increased domestic physical activity, i.e., general housework, such as washing dishes, washing and ironing clothes, and similar activities.

### Intensity of Physical Activity

During the coronavirus pandemic in Brazil, it is recommended that the intensity of physical exercise be light to moderate, given that very high intensity may lead to more accentuated immunosuppression, requiring longer recovery time.

### Duration of Physical Activity

During the coronavirus pandemic in Brazil, it is recommended that the duration of each exercise session be approximately 30 to 60 minutes daily. It is suggested that total time not be too long due to the immune system depression with longer recovery time.

### Suggestions for reducing sedentary behavior during the novel coronavirus pandemic

Considering that, in addition to regular practice of physical activity, it is very important to reduce sedentary behavior, the following are recommended:

1. Reduce sedentary behavior to a maximum of 6 to 8 cumulative hours daily.
2. Reduce to a maximum of 2 to 4 hours sitting in front of the screen daily.
3. Attempt to maximize the number of interruptions/pauses to sitting time, namely, for every hour spent sitting, stand up for at least 5 minutes.

## Final considerations

Based on the studies consulted, the evidence confirms the importance of continuing to practice physical activities during the novel coronavirus pandemic, with light to moderate intensity and duration, preferably in outdoor environments or at home. In addition, it is also very important to emphasize reducing sedentary behavior, namely, time spent sitting, lying down, or reclining, excluding sleep hours, and time spent in front of the television, computer, and similar devices.
